# Determinants of the Cost-Effectiveness of Intermittent Preventive Treatment for Malaria in Infants and Children

**DOI:** 10.1371/journal.pone.0018391

**Published:** 2011-04-07

**Authors:** Amanda Ross, Nicolas Maire, Elisa Sicuri, Thomas Smith, Lesong Conteh

**Affiliations:** 1 Swiss Tropical and Public Health Institute, Basel, Switzerland; 2 University of Basel, Basel, Switzerland; 3 Barcelona Centre for International Health Research (CRESIB), Hospital Clínic-Universitat de Barcelona, Barcelona, Spain; 4 CIBER Epidemiología y Salud Pública (CIBERESP), Barcelona, Spain; 5 Institute of Global Health Innovation, Imperial College London, London, United Kingdom; Universidade Federal de Minas Gerais, Brazil

## Abstract

**Background:**

Trials of intermittent preventive treatment in infants (IPTi) and children (IPTc) have shown promising results in reducing malaria episodes but with varying efficacy and cost-effectiveness. The effects of different intervention and setting characteristics are not well known. We simulate the effects of the different target age groups and delivery channels, seasonal or year-round delivery, transmission intensity, seasonality, proportions of malaria fevers treated and drug characteristics.

**Methods:**

We use a dynamic, individual-based simulation model of *Plasmodium falciparum* malaria epidemiology, antimalarial drug action and case management to simulate DALYs averted and the cost per DALY averted by IPTi and IPTc. IPT cost components were estimated from economic studies alongside trials.

**Results:**

IPTi and IPTc were predicted to be cost-effective in most of the scenarios modelled. The cost-effectiveness is driven by the impact on DALYs, particularly for IPTc, and the low costs, particularly for IPTi which uses the existing delivery strategy, EPI. Cost-effectiveness was predicted to decrease with low transmission, badly timed seasonal delivery in a seasonal setting, short-acting and more expensive drugs, high frequencies of drug resistance and high levels of treatment of malaria fevers. Seasonal delivery was more cost-effective in seasonal settings, and year-round in constant transmission settings. The difference was more pronounced for IPTc than IPTi due to the different proportions of fixed costs and also different assumed drug spacing during the transmission season. The number of DALYs averted was predicted to decrease as a target five-year age-band for IPTc was shifted from children under 5 years into older ages, except at low transmission intensities.

**Conclusions:**

Modelling can extend the information available by predicting impact and cost-effectiveness for scenarios, for outcomes and for multiple strategies where, for practical reasons, trials cannot be carried out. Both IPTi and IPTc are generally cost-effective but could be rendered cost-ineffective by characteristics of the setting, drug or implementation.

## Introduction

An estimated 250 million episodes of malaria led to nearly one million deaths in 2008, the brunt of which was borne by young children and infants in sub-Saharan Africa [1]. In addition to its impact on the health of individuals, malaria places considerable costs on households [2]–[4], communities [5] and nations [6], [7].

Intermittent preventive treatment in infants (IPTi) and children (IPTc) have received attention in recent years as potential interventions to reduce malaria morbidity and mortality. Both follow the same strategy: to deliver a full course of an anti-malarial treatment to a population at risk at specified time points whether or not they are known to be infected [8], [9]. Both aim to retain the benefits of chemoprophylaxis whilst avoiding the acceleration of drug resistance or impairing the development of acquired immunity [9]–[11].

The two interventions differ in their target age group and delivery system. By targeting infants under 12 months, IPTi is able to benefit from the existing delivery strategy of the Expanded Programme on Immunization (EPI). The delivery of IPTi involves training health workers to administer a dose of an antimalarial drug during routine scheduled visits in health facilities and to document this using modified EPI monitoring tools [12], [13]. IPTc has targeted mainly children up to the age of five years [14]–[22] but also older age groups [23] and school children [24]–[27]. In children under five years, IPTc has no established delivery system but studies have used community health workers and outreach clinics to provide doses for the target age-group [20], [21]. Studies have mostly focused on IPTc as a seasonal intervention in settings with seasonal transmission.

Both IPTi and IPTc have been found to reduce clinical incidence. Several clinical trials in different settings have shown IPTi to be effective against malaria to varying degrees [28]–[33]. A pooled analysis of data from six completed trials of IPTi with sulfadoxine-pyrimethamine (SP) estimated a 30% (95% CI 20%, 39%) protective efficacy (PE) against clinical malaria and 38% (13%, 56%) PE against hospital admissions with malaria parasites [34]. Studies using drugs other than SP found that efficacious, long-lasting drugs had a greater PE than shorter-acting drugs or drugs with high levels of resistance [35], [36]. Seasonal IPTc with combinations of SP, artesunate (AS) and amodiaquine (AQ) has been seen to reduce the incidence of clinical malaria in children under the age of five years in settings with a short malaria transmission season [14], [15], [17], [37] and where the transmission season is longer [19], [38]. A pooled analysis of IPTc trials estimated a 75% (64%, 83%) protective efficacy against malaria episodes during the intervention period [39]. The trials were not designed to detect an impact on mortality due to the very large sample sizes required.

There have been costing and cost-effectiveness studies alongside many of the IPT trials [12], [13], [21], [40]–[42]. In nearly all of the studies where IPT was efficacious, it was highly cost-effective. In sites where IPTi had a significant effect, the cost per malaria episode averted for IPTi-SP ranged from US$ 1.36 to 4.03 based on trial specific data [40]. For IPTi using more expensive antimalarials, the cost per treated episode averted ranged from US$4.62 using AQAS to US$ 18.56 using mefloquine (MQ) [40]. For seasonal IPTc, a trial in Ghana estimated the costs per episode averted for three different regimens administered over the six month transmission period. Bimonthly SP cost $105 ($75, $157) per treated episode averted, bimonthly ASAQ was $212 ($127, $399) and monthly ASAQ was US$68 ($62, $75) [42]. The estimates for district scale-up fell to $28, $60 and $22 respectively [42]. In addition, where efficacious, IPT reduced health system costs and showed significant savings to households from malaria episodes averted.

The variations in efficacy and cost-effectiveness between trials stem not only from the choice of drug but also from the different setting and trial characteristics such as transmission intensity, timing of delivery, local costs and use of other interventions. This raises questions that the trials were not designed to answer such as the effects of the different characteristics, the impact on severe malaria and mortality, and the limits beyond which IPT is no longer cost-effective [43]–[47]. It is not feasible to carry out a large number of large field trials of different combinations to determine the impact of each factor on different malariological outcomes. Where data cannot be collected, mathematical modelling can be used to provide predictions.

In this paper, we use a comprehensive model of *Plasmodium falciparum* epidemiology and economics [48] to investigate the influence of different variables on the effects and the cost-effectiveness of IPT in preventing disability adjusted life years (DALYs): target age group and delivery channel, seasonal or year-round delivery, transmission intensity and seasonality of the setting, the timing of the first IPT dose for seasonal delivery, different coverage levels of treatment for malaria fevers and the impact of drug resistance. We also predict the impact of IPT on transmission intensity.

## Methods

### The simulation model

We use a dynamic, individual-based, stochastic simulation of malaria epidemiology which has been described elsewhere [48]. Briefly, there is a simulated population of humans who are updated at each five-day time step via model components representing new infections, parasite densities, acquired immunity, uncomplicated and severe episodes, direct and indirect malaria mortality, infectivity to mosquitoes and case management. This study does not include simulation of anaemia. The course of parasite densities over an infection are described by averaged empirical data [49]. Immunity to asexual parasites is derived from a combination of cumulative exposure to both inoculations and parasite densities, and maternal immunity [49]. The inclusion of acquired immunity allows us to model potential effects of IPT on immunity through loss of exposure and the inclusion of infectivity captures potential effects on transmission intensity. The probability of a clinical attack of malaria depends on the current parasite density and a pyrogenic threshold [50]. The pyrogenic threshold responds dynamically to recent parasite load, increasing or saturating with exposure to parasites and decaying with time, and thus is individual-and time- specific. Severe malaria can arise in two ways, either as a result of overwhelming parasite densities or through uncomplicated malaria with concurrent non-malaria co-morbidity [51]. Mortality can be either direct (following severe malaria) or indirect (uncomplicated malaria in conjunction with co-morbidity, or during the neonatal period as a result of maternal infection). Thus the model does not assume a fixed case fatality rate for malaria episodes, but makes a number of intermediary assumptions to model pathways from an acute episode to death. The parameter values for each of the components of the model were estimated by fitting to data from a total of 61 malaria field studies of different aspects of malaria epidemiology [52] and are reported elsewhere [53]. The model has been validated using age-specific results from six IPTi trials with SP [53]. It has subsequently been validated against trials of IPTc [17], [19].

### Simulation strategy

We simulate seasonal and non-seasonal delivery for both IPTi and IPTc to allow us to separate the effects of seasonal delivery from the combination of the intervention target age-group and delivery channel. We simulate two contrasting seasonal patterns (constant and highly seasonal transmission) and two IPT drugs (SP and ASAQ). These four factors have two levels each making a set of 16 baseline intervention scenarios (Table 1). For each of these scenarios, we then investigated the impact of varying levels of drug resistance, transmission intensity, the timing of seasonal implementation and the proportion of malaria fevers which are effectively treated (Table 2). We also simulate the impact of either widening the target IPTc age group or shifting it into older ages. We simulate 10 seeds for each scenario each of a population of 100,000 individuals aged up to 90 years over ten years from the introduction of the IPT programme.

**Table 1:** Set of baseline intervention scenarios.

Variable	Levels	IPTi	IPTc
Target ages		3, 4 and 9 months	3 months to 5 years
Period of delivery	Year-round	At 3, 4 and 9 months through EPI	Bi-monthly doses via CHW
	Seasonal	At 3, 4 and 9 months if EPI visits coincide with malaria season	Monthly doses for 3 months via CHW
IPT coverage per dose		95%	84%
IPT drug		SP	SP
		ASAQ	ASAQ
Seasonality of setting	No seasonality	Constant transmission	Constant transmission
	Highly seasonal^1^	Niakhar	Niakhar

EPI: Expanded Programme on Immunization CHW: community health workers.

1 The highly seasonal pattern of transmission was taken from Niakhar, Senegal [89] where transmission is concentrated into three months of the year.

**Table 2:** Model inputs varied in the simulations.

Variable	Description	Levels
Transmission intensity	Infected bites per person per year prior to the introduction of IPT	1, 6, 21 (baseline), 50
Case-management coverage	Proportion of fevers treated per 5 day timestep	0, 4 (baseline), 10, 30, 50, 75 and 100%
Timing of seasonal delivery of IPT	In seasonal setting only	The period of delivery is shifted up to 4 months earlier or later than the baseline scenarios (Figure 1)
Frequency of drug resistance	Percentage of wildtype infections	SP*: 0, 20, 40, 60, 80 (baseline), 100%
		ASAQ: 90% wildtype, 10% fully resistant to AQ
Target ages for IPTc	Widening age groups	3 m–5 y, 3 m–10 y, 3 m–15 y,…3 m–35 y,3 m–40 y
	Shifting age groups	3 m–5 y, 2.5–7.5 y,5–10 y,7.5–12.5 y,…35–40 y

*For SP, the remainder is divided 50:50 between single/double and triple *dhfr* mutations.

### Delivery frequency and modality of IPTc and IPTi in the baseline scenarios

For IPTi, the EPI visits were assumed to be scheduled at 3, 4 and 9 months of age (Table 1). For seasonal delivery, only infants who were presented for their EPI visits during the short transmission season received the doses and so no infant would receive all three doses.

**Figure 1:**
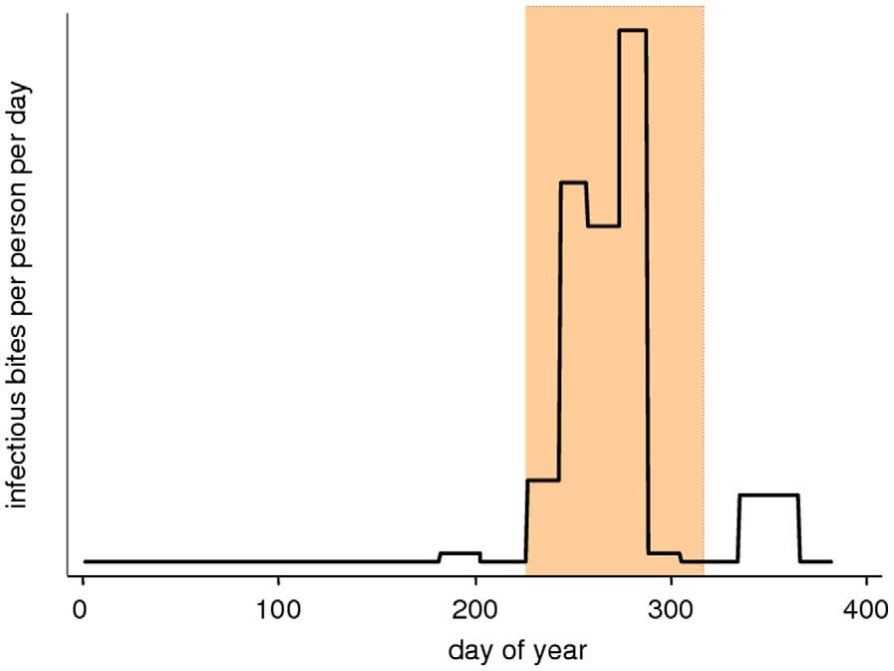
Timing of seasonal delivery in the baseline scenarios. The shaded area is the 3 month period of IPT delivery. The seasonal pattern follows that reported for Niakhar, Senegal [89].

The baseline target IPTc age group was 3 months to 5 years. We model IPTc delivery either every two months throughout the year or as three IPTc doses at monthly intervals during the malaria season. IPTc was assumed to be delivered, and costed, via community health workers (CHWs) who were individuals in the community given a small financial incentive to deliver IPTc. Studies in The Gambia and Ghana found that CHWs were able to reach more children under five years than outreach services linked to EPI [20], [21]. The timing of the doses relative to the start of the season for seasonal delivery is shown in Figure 1. In the baseline scenarios, we assumed that IPTi coverage was 95% per dose (86% for 3 doses) [53] and that IPTc coverage was 84% per dose for CHW [20], [21]. ASAQ includes 3 tablets to be taken on 3 consecutive days: we assumed that compliance with the tablets given to carers to administer at home was 100%. Compliance has been reported to be 88% to 99% in trial settings [18], [20], [22], [38].

### IPT drugs

We simulate two drugs, ASAQ and SP, both of which have previously been chosen for IPT trials. They demonstrate different characteristics: SP is a cheap, long-acting drug with high levels of resistance whereas ASAQ is a more expensive, shorter-acting drug currently meeting less drug resistance. We recognise that there are other potential IPT drug candidates that we have not included, nevertheless, the contrasting characteristics of these two drugs demonstrate the substantive effects.

A simple model component for antimalarial drug action [53] was adapted from Hastings and Watkins [54] and incorporated into the simulations. Briefly, the ability of SP and ASAQ to both clear existing infections and to prevent new infections becoming established depends on the genotype of the infection. Infections are assigned genotypes randomly according to assumed frequencies (Table 2). Hastings and Watkins quantify the chances of failing treatment with correct dosing of SP for wildtype infections and infections with single, double and triple dihydrofolate reductase (*dhfr*) mutations conferring resistance at 0, 0, 0, 50% respectively while periods of prophylactic effects are 52, 12, 12, 2 days [54]. We round these to the five-day timesteps used by the model. For ASAQ, we assume that all infections are cleared and that for AQ-sensitive infections, the prophylactic period is assumed to be 15 days and for AQ-resistant infections, 0 days [55]–[57].

### Intervention costs

The costs (Table 3) were based on economic evaluations conducted in a range of IPTi and IPTc sites [13], [40], [42], [58]. Similar cost categories and methodological costing approaches were used for both interventions covering district costs associated with community sensitization, behaviour change and communication, drug distribution and administration, training and supervision. The costs were identified from components of trial budgets and primary data on resource use. Care was taken to exclude costs relating specifically to research or to a trial environment. We costed IPTc only in the baseline target age group of 3 months to 5 years since while schools may be used for some age groups [26], the delivery mechanisms for others are unclear. Costs of incentivising the CHW who delivers the IPTc drugs to an assumed 250 children were included in administration costs to reflect an allowance of approximately US$10 a month during the months of administration [20], [21]. The costs of the IPT drugs were based on prices presented on the International Drug Price Indicators List [59]: SP was assumed to cost $0.02 per dose and ASAQ $0.36 per course of three tablets. Remainder fractions of tablets were assumed to be wastage. The cost of the intervention to households was found to be negligible and therefore excluded [21]. The cost per dose of IPT drugs, delivery and administration remained unchanged for each dose irrespective of the number of doses given. The costs of training, sensitization and a minimum level of supervision were assumed to be fixed over the course of the year and therefore the unit cost per dose of IPT year-round was less than that of seasonal delivery. This assumption was based on trial activity and discussions with implementers about how IPT would be delivered if introduced as part of routine activity. Whether the doses were given throughout the year or concentrated in three months, a one-off training would be held each year for those involved in delivering IPT. Sensitization activities would involve the same resources even though the message about IPT frequency would be different. Supervision was assumed to be semi-fixed in that a minimum would be required and so it would be slightly more intense for seasonal than year-round IPT. The costs were calculated in US$2006 to be comparable with previous costs for case management [60], all costs were then inflated to US$2009 using US dollar inflation rates [61].

**Table 3:** Unit costs per dose (USD 2009).

	IPTc^1^				IPTi			
	seasonal		year-round		seasonal		year-round	
	SP	ASAQ	SP	ASAQ	SP	ASAQ	SP	ASAQ
Cost of IPT drugs	0.02	0.36	0.02	0.36	0.02	0.36	0.02	0.36
Drug dispensing and supplies	0.20	0.20	0.20	0.20	0.01	0.01	0.01	0.01
Delivery of drugs	0.09	0.09	0.09	0.09	0.06	0.06	0.06	0.06
Supervision	0.21	0.21	0.15	0.15	0.01	0.01	0.01	0.01
Training	0.14	0.14	0.07	0.07	0.13	0.13	0.04	0.04
Sensitization	0.03	0.03	0.02	0.02	0.03	0.03	0.01	0.01
Total	0.69	0.97	0.56	0.90	0.27	0.61	0.16	0.50

1 IPTc for baseline age group of 3 months to 5 years using village health workers.

### Potential cost savings of IPT

The simulations include direct malaria treatment costs to both the providers and households. We do not include indirect costs such as potential earnings forgone by the carers [60]. The health system adopted is based on a previously used model [62] with artemisinin combination therapy as the first-line treatment with low rates of access. The case management costs assumed have been previously published [60].

### Cost-effectiveness

The approach adopted follows previous work on modelling the cost-effectiveness of malaria vaccines [60], [63] and follows standard practices [64], [65]. The primary epidemiological outcome was the number of DALYs averted since they are a comparable, summary measure of the burden. One DALY represents a year of healthy life lost. Years of life lived with disability were calculated on the basis of the duration of disability and the disability weights for the different malaria attributable disease conditions obtained from the Global Burden of Disease study [66]. DALYs were calculated assuming age-specific life expectancies typical for an East African setting with low malaria transmission [60], [63] and with no age weighting to follow standard cost-effectiveness practices [67]. Future costs and health gains are discounted at 3%. The cost-effectiveness ratios are to be interpreted as incremental cost-effectiveness ratios (ICERs) of implementing the interventions in the simulated scenarios relative to a do nothing scenario which corresponds to maintaining only case management.

Recognising that the selection of cost-effectiveness thresholds in published literature is subjective [68], we refer to the conservative cut off point of US$ 223 per DALY averted to reflect a cost-effective intervention, and US$ 37 per DALY averted to reflect a *highly* cost-effective intervention. These thresholds are based on US$ 150 and US$ 25 thresholds suggested by the World Bank in 1993 [68], [69] and inflated to their 2009 equivalent.

## Results

Both IPTc and IPTi were cost-effective in a wide range of simulated settings.

### Seasonal and year-round delivery in seasonal and perennial transmission settings

**Figure 2:**
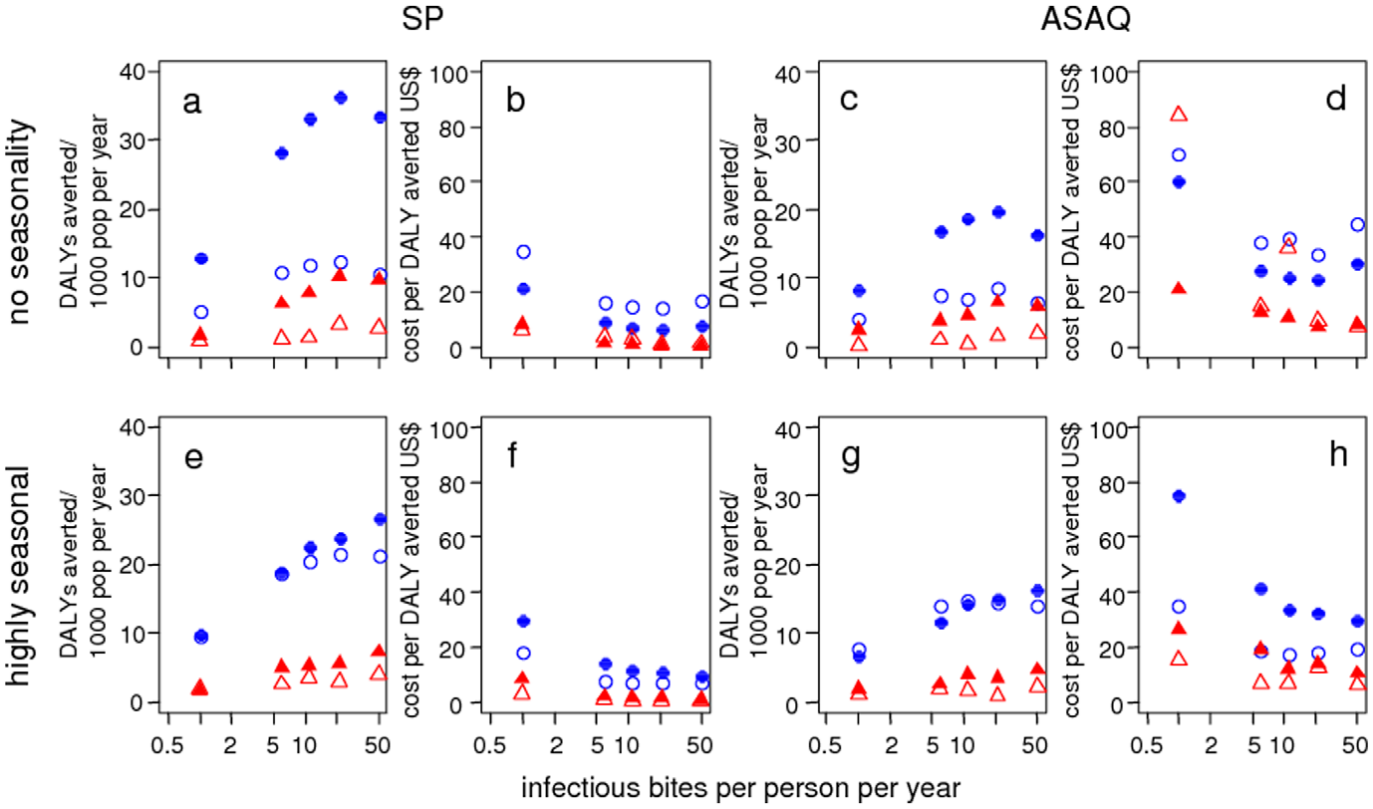
Predicted number of DALYs averted and cost per DALY averted by transmission intensity. Blue filled circles: year-round IPTc in children aged 3 months to 5 years, blue hollow circles: seasonal IPTc, red filled triangles: year-round IPTi, red hollow triangles: seasonal IPTi. Top row (constant transmission): a) DALYs averted and b) cost per DALY averted for IPT with SP c) DALYs averted and d) cost per DALY averted for IPT with ASAQ. Bottom row (Niakhar seasonality): e) DALYs averted and f) cost per DALY averted for IPT with SP, g) DALYs averted and h) cost per DALY averted for IPT with ASAQ. The baseline scenario values are given in Tables 1 and 2. Simulated individuals are aged 0–90 years. There is a large degree of stochasticity in costs per DALY where few DALYs are averted.

The effect of seasonal delivery depends on the seasonal pattern of transmission. In the constant transmission setting (Figure 2 top row), year-round delivery averts a greater number of DALYs than seasonal delivery for both IPTi and IPTc (Figure 2 a and c). Year-round delivery is also more cost-effective than seasonal delivery, substantially so for IPTc whereas for IPTi the difference is less pronounced. As well as the different unit costs, the different spacing of doses in combination with the assumed prophylactic periods contribute to year-round IPTc delivery being more cost-effective. For IPTi, the numbers of DALYs averted are proportional to the numbers of doses administered.

In the highly seasonal setting (Figure 2, bottom row), the numbers of DALYs averted are much closer for seasonal and year-round delivery since there are few episodes outside the main transmission season. In this case, seasonal delivery is more cost-effective than year-round for both IPTi and IPTc due to the lower total costs.

### Transmission intensity and target age group

We focus on the lower end of the range of transmission because interest lies in determining where IPT is not cost-effective (Figure 2). For all of the IPT strategies, the predicted number of DALYs averted is low at low transmission intensities and increases up to a plateau at moderate levels decreasing slightly at very high transmission intensities. This slight decrease in DALYs averted at high transmission intensities occurs despite increasing predicted total numbers of DALYs in the scenarios with no IPT. The predictions suggest that IPTi and IPTc are cost-effective, although not highly cost-effective, at the low transmission intensities simulated. With zero transmission, IPT would clearly not be cost-effective. A clear lower limit below which IPT is not cost-effective is not obvious: care should be taken with interpretation since the model was created using data mainly from medium and high transmission settings and so there is greater uncertainty in predictions for low transmission intensity settings. The predictions do however indicate an approximately log-linear relationship.

**Figure 3:**
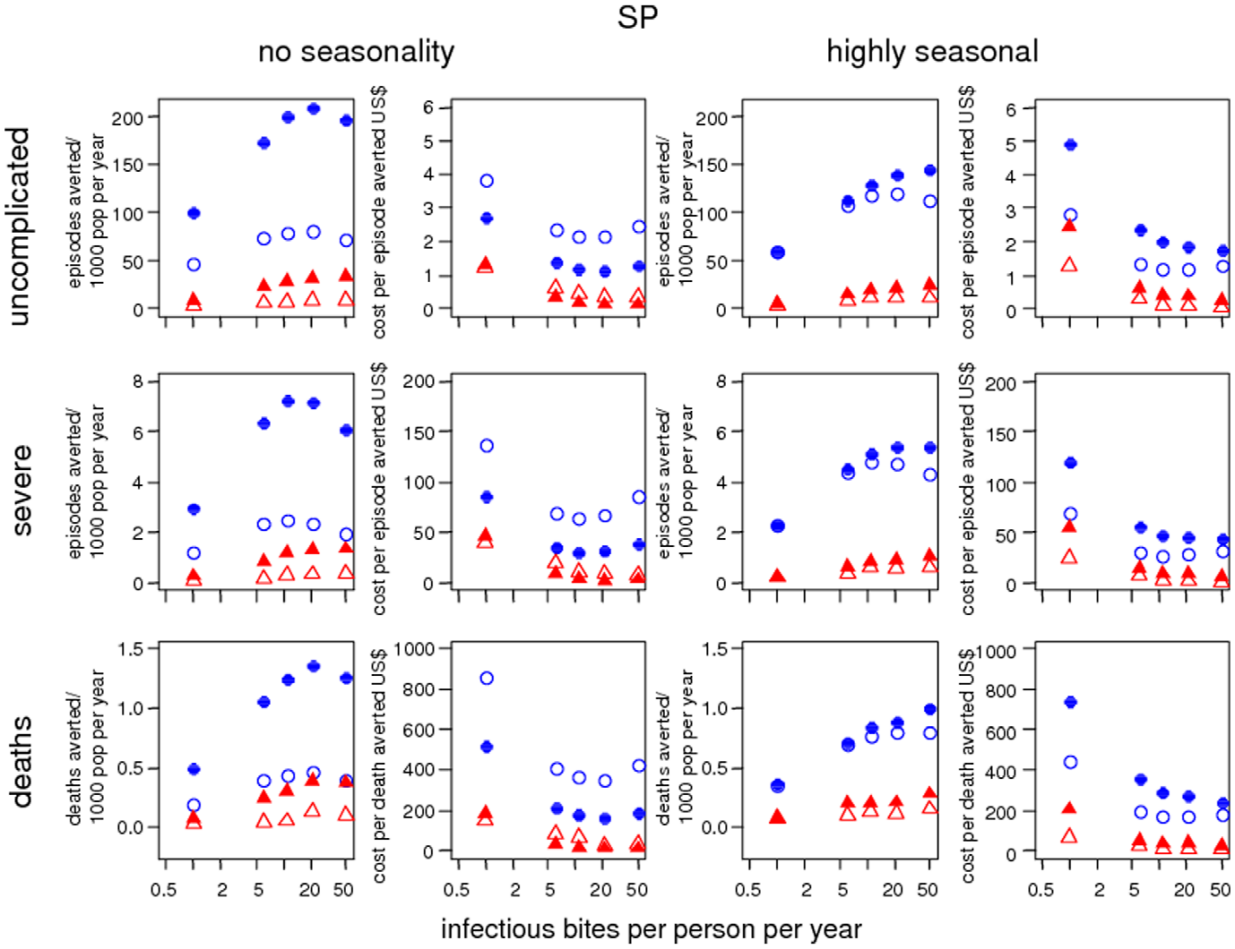
Predicted number of episodes averted and cost per episode averted by transmission intensity. Blue filled circles: year-round IPTc in children aged 3 months to 5 years, blue hollow circles: seasonal IPTc, red filled triangles: year-round IPTi, red hollow triangles: seasonal IPTi. The baseline scenario values are given in Tables 1 and 2. Simulated individuals are aged 0–90 years.

Since the age-distribution of episodes is affected by transmission intensity [45], [70], the relative impact at a low transmission intensity was compared for IPTc and IPTi for uncomplicated episodes, severe episodes and deaths (Figure 3). IPTc averts more clinical events than IPTi in the simulated scenarios, and the ratio of clinical events averted by IPTc to IPTi is greater at an EIR of 1 than at an EIR of 21. The size of the ratio, however, depends on the outcome and the seasonality of the setting.

**Figure 4:**
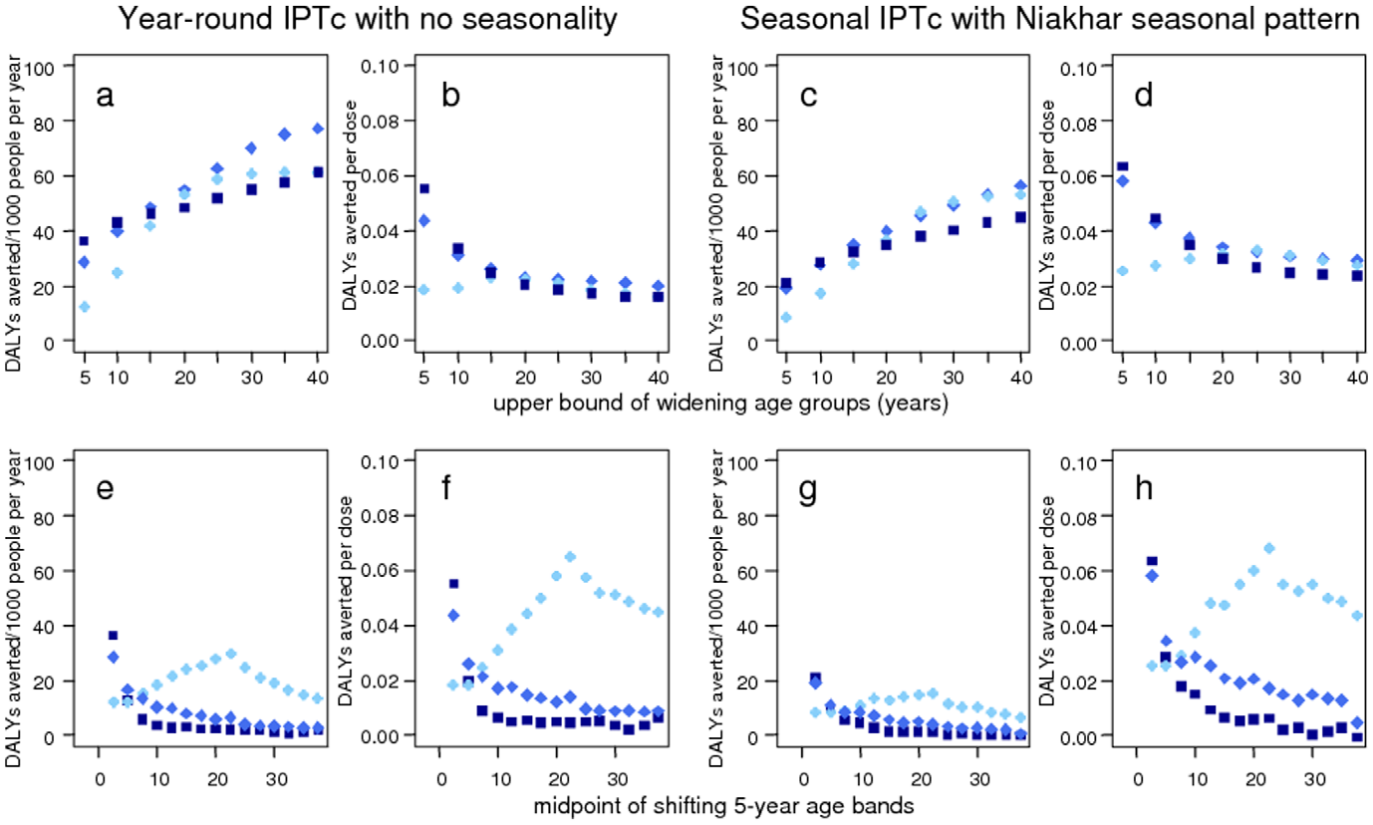
Predicted number of DALYs averted and DALYs averted per dose by IPTc target age group. Light blue circles  =  EIR 1; mid-blue diamonds =  EIR 6; dark blue squares =  EIR 21. Top row (widening age groups): DALYs averted and DALYs averted per dose for constant seasonality with year-round IPTc a) and b), and Niakhar seasonality with seasonal IPTc c) and d). Bottom row: shifting five-year age-bands.

The predicted number of DALYs averted increased as the target age group for IPTc was widened to include older children for all transmission levels simulated (Figure 4, top row), although the number of DALYs averted per IPT dose decreased. Since the predictions cover the first ten years of an IPT programme they will not include potential rebound effects in those receiving IPT for long periods of time. Increasing the target ages of a five-year wide age-band for IPTc (Figure 4 bottom row) lead to a decrease in DALYs averted with increasing age for settings with an EIR of 6 and 21. The scenario with an EIR of 1, however, increased gently to a maximum at 20 years. This prediction is highly uncertain, and is driven in the model by the increased surface area of the individual leading to a higher number of mosquito bites. Nevertheless, the predictions suggest that IPT would be more beneficial in older age groups at very low transmission intensities.

### Choice of IPT drug

In all of our simulated scenarios, SP averted a greater number of DALYs and was more cost-effective than ASAQ. This is driven by the longer prophylactic period of SP, the lower costs and the low levels of drug resistance we have assumed.

### Timing of first dose in seasonal settings

**Figure 5:**
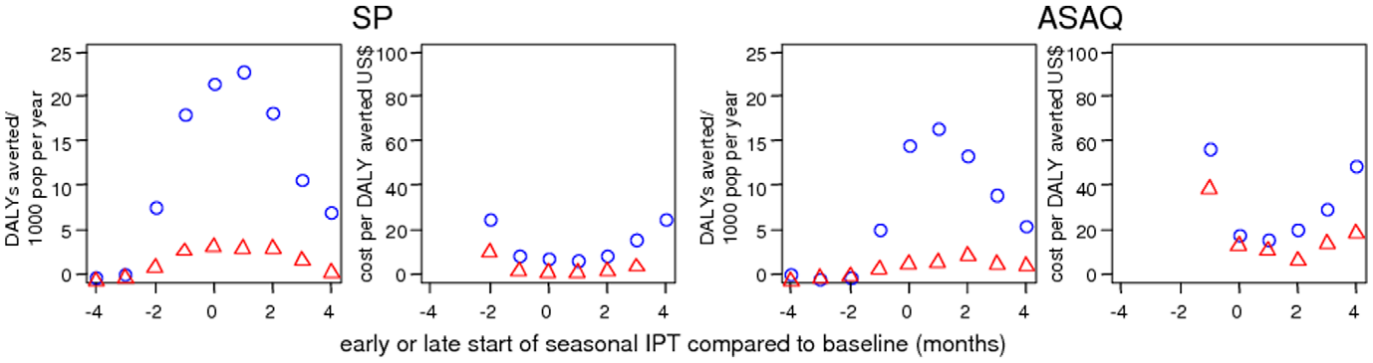
Predicted effects and cost-effectiveness of IPT depending on timing of first dose in seasonal settings. Blue hollow circles: seasonal IPTc in children aged 3 months to 5 years, red hollow triangles: seasonal IPTi. Very high and low costs per DALY were not included for early implementations where the number of DALYs averted were very low since these values were subject to considerable stochasticity. The baseline scenario values are given in Tables 1 and 2. Simulated individuals are aged 0–90 years.

The cost-effectiveness of seasonal delivery of IPTi and IPTc in a highly seasonal setting is sensitive to the timing of the first dose (Figure 5). Too early or too late and at least part of the treatment and prophylactic actions of the drug are wasted. There is some leeway however and SP was less sensitive to timing than ASAQ due to the longer prophylactic period. If the three-month delivery period was begun very early, very few DALYs were averted and the corresponding costs per DALY were subject to considerable stochasticity: these values could not be included on the figures. Whilst the extent of mistiming is unlikely to be three months in practice, these predictions show that badly timed implementation can push IPT over the cost-effectiveness threshold.

### Proportions of malaria fevers treated

**Figure 6:**
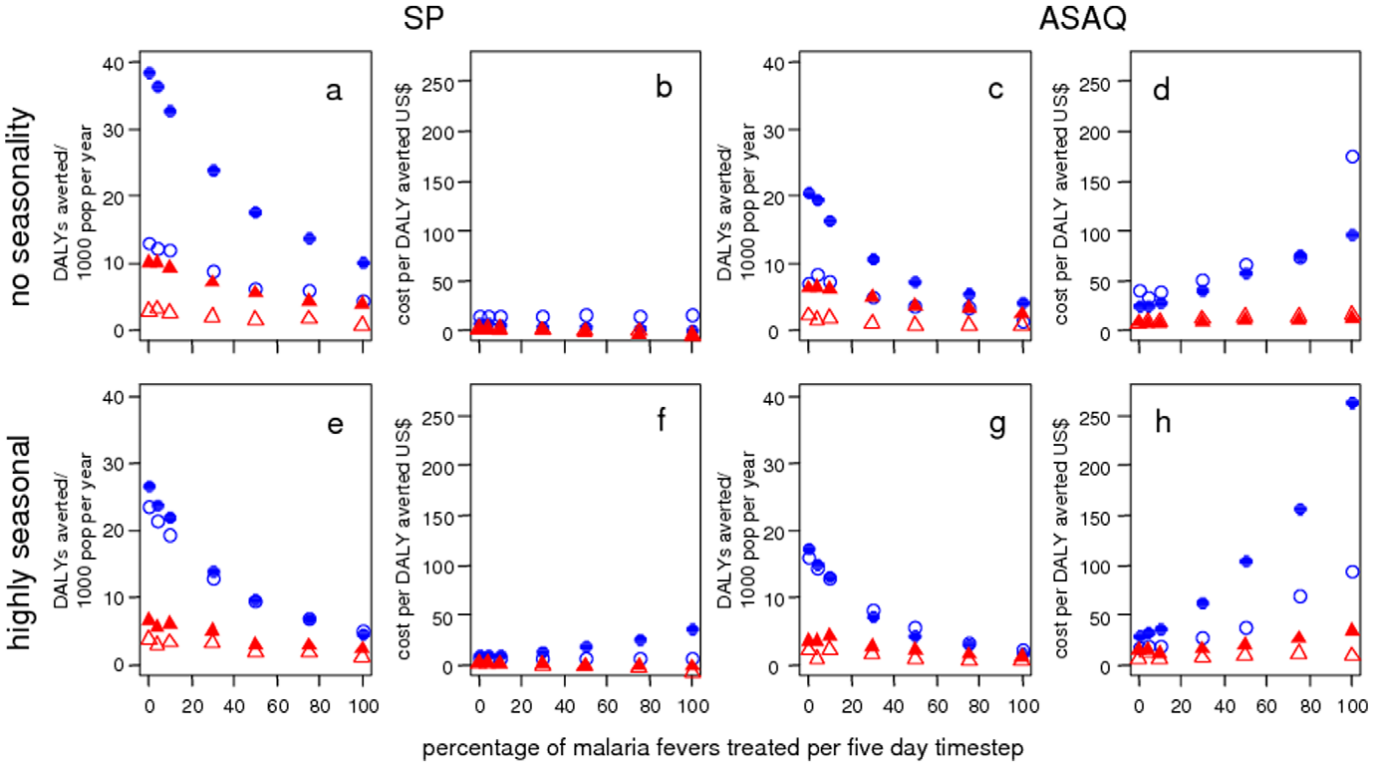
Predicted number of DALYs averted and cost per DALY averted by case-management coverage. Blue filled circles: year-round IPTc in children aged 3 months to 5 years, blue hollow circles: seasonal IPTc, red filled triangles: year-round IPTi, red hollow triangles: seasonal IPTi. Top row (constant transmission): a) DALYs averted and b) cost per DALY averted for IPT with SP c) DALYs averted and d) cost per DALY averted for IPT with ASAQ. Bottom row (Niakhar seasonality): e) DALYs averted and f) cost per DALY averted for IPT with SP, g) DALYs averted and h) cost per DALY averted for IPT with ASAQ. The baseline scenario values are given in Tables 1 and 2. Simulated individuals are aged 0–90 years.

As treatment coverage increases the number of DALYs averted by IPT decreases and the costs per DALY increase (Figure 6). This is driven by a reduction in the total DALY burden: the prompt treatment prevented severe malaria and deaths and cleared infections which could later produce symptoms and the high treatment coverage of all age groups lead to a small reduction in transmission. There was no apparent synergy between health system coverage and IPT. At very high treatment coverage levels using ASAQ, some scenarios are no longer cost-effective. However, this only occurred at treatment levels which would be unrealistic even for very good health-systems.

### Drug Resistance

**Figure 7:**
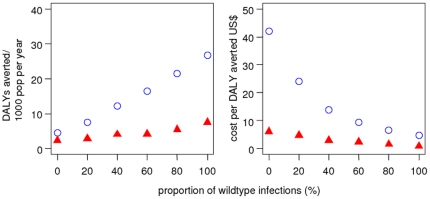
Predicted impact and cost-effectiveness of IPT by SP drug sensitivity. Blue hollow circles: seasonal IPTc, red filled triangles: year-round IPTi. SP sensitivity is quantified as the proportion of wildtype infections with no *dhfr* nutations, with the remainder being divided 50:50 between infections with *dhfr* single or double and triple mutations. The action of SP varies according to these genotypes (Methods section). The baseline scenario values are given in Tables 1 and 2. Simulated individuals are aged 0-90 years.

We simulated the effect of varying levels of drug resistance for year-round IPTi and seasonal IPTc both with SP only. In both cases, the number of DALYs averted decreased with rising drug resistance and the corresponding cost-effectiveness decreased. In all of the scenarios simulated, IPT remained cost-effective. However, if a drug has no effect whatsoever, then clearly this would not be the case [35], [71]. We did not simulate levels of resistance which would render SP completely ineffective. The linear pattern between DALYs averted and drug sensitivity (Figure 7a) was also observed for constant seasonality and for other transmission intensities with an annual EIR of 1 and 200 (not shown).

### Impact of IPTi and IPTc on transmission intensity

We predicted a negligible impact of both IPTi and IPTc on the infectious reservoir and on transmission intensity except where the wider age groups for IPTc were simulated.

## Discussion

The predictions suggest that both IPTi and IPTc are cost-effective in the majority of scenarios simulated, even with the conservative thresholds we have used. In general, IPTc averted a greater number of DALYs than IPTi, but the costs were greater and consequently the costs per DALY averted were greater. A greater number of DALYs are averted for both IPTc and IPTi by year-round compared to seasonal delivery in perennial transmission settings, but similar numbers of DALYs are averted in seasonal settings since there are few episodes outside the main transmission season. Seasonal delivery is more cost-effective in seasonal settings, and year-round in constant transmission settings. However the difference is more pronounced for IPTc than IPTi due to the different proportions of fixed costs and also different assumed drug spacing during the transmission season. Cost-effectiveness was predicted to decrease with decreasing transmission, badly timed seasonal delivery in a seasonal setting, short-acting and more expensive drugs, increased frequencies of drug resistance and increasing levels of treatment coverage. A greater number of DALYs were averted as the target age groups were widened to include older children for IPTc in all simulated transmission settings, although the number of DALYs averted per IPT dose fell slightly. The number of DALYs averted decreased as the target ages for a five-year age-band were increased except for very low transmission intensities. This concurs with a systematic review of the age-distribution of episodes by transmission intensity in children [70], however the burden in adults is not well established.

Our aim was not to pit IPTc and IPTi against each other since they are both interventions focusing on drug administration and differing only in target age group and delivery system. Instead, we aimed to tease out the contribution of seasonal delivery in different settings for both interventions and to investigate factors which affect their impact and cost-effectiveness. We selected a limited number of scenarios in order to focus on a manageable number of questions and to investigate the substantive effects. We recognize that there are many other potential scenarios differing in characteristics such as IPT schedule, target age groups, candidate drugs, seasonal patterns and also equity and heterogeneity in IPT coverage [45].

The costs of an IPT programme are largely driven by the cost of the IPT drug and, in the case of IPTc, delivery since it does not benefit from the existing delivery strategy EPI. The predicted cost- effectiveness is driven by the low costs, particularly for IPTi, and the impact on DALYs, particularly for IPTc. The DALYs were dominated by the contribution from deaths.

Our predicted costs per episode averted are generally lower than estimates from clinical trials. The predicted cost per uncomplicated episode averted ranges from $1.08 to 17.59 for seasonal IPTc including all the values for transmission intensity (Figure 3), drug resistance and treatment coverage in comparison to $22 to $60 per treated episode estimated for district delivery from trial data [42]. For year-round IPTi with SP, the predicted cost per episode averted ranges from $0.42 to $7.71 (Figure 3 and not shown) compared to $1.36 to $11.93 per treated episode from trial studies [40]. Although our predictions are not of the specific trials and so differ in many ways, the largest single reason for the difference is that we have predicted all episodes whereas the trial data refers to treated episodes only. Our model for acute episodes [50] is fitted to data from Ndiop and Dielmo in Senegal where intensive daily active surveillance was carried out [72] thus capturing episodes unavoidably missed by passive case detection.

The predicted cost-effectiveness of IPTc and IPTi is in line with other malaria control interventions. Inflating using US dollars only to US$ 2009, the cost per DALY averted of insecticide-treated net programmes is estimated to range from $14 to $74 [73]–[75], and for indoor residual spraying, US$131–145 [73]. Case management is estimated to cost between $11 and $31 depending on the treatment drug and for IPTp estimates vary from $42 to approximately $422 per DALY averted [74], [75].

The question of where the boundaries of IPT cost-effectiveness lie has been raised, particularly for transmission intensity [43], [44]. Our predictions show that, as transmission intensity decreases to low levels, the number of DALYs averted decreases and the corresponding cost per DALY increases. Unfortunately, while the log-linear nature of the relationship is apparent from our predictions, a boundary where IPTc and IPTi are no longer cost-effective is not. Even at low transmission intensities, IPT is predicted to be cost-effective although not highly cost-effective according to the World Bank thresholds however IPT would clearly not be cost-effective if there was no transmission. Caution must be taken with interpreting the predictions for settings with low transmission intensities since the model was created used data from mainly medium and high transmission settings and does not take into account variables such as heterogeneity in transmission or decay of immunity which may have strong effects in low transmission settings [52]. In addition, children under five years old are not predicted to be the optimum target group at very low transmission intensities and the combination of other factors is important, for example higher frequencies of drug resistance would increase the levels of transmission intensity at which IPT is no longer cost-effective.

Both IPTi and IPTc in children under five years of age were predicted to have negligible effects on transmission in this study. This agrees with previous predictions of IPT in these target age groups [53], [76] although simulations of IPTc in children aged 5 to 18 years suggested a reduction in transmission [77]. These results indicate that a wider target age range including older children would be necessary to reduce transmission, such as is being considered for mass screen and treat.

An increased incidence of clinical episodes following the end of the prophylactic period was observed in some IPTi trials, but not others [34]. In the seasonal IPTc trials which followed the participants up over the following season, no rebound effects were observed in individual trials [17], [19], [23] but a meta-analysis indicated a small increase in the incidence of clinical episodes [39]. A previous study of year-round IPTi predicted a modest increase in susceptibility following the prophylactic period which was outweighed by the cumulative benefits [53]. The model would also predict this same pattern for IPTc doses in older children, although to a much lesser extent if there is a seasonal break in transmission. Monthly SP may be akin to chemoprophylaxis [44] after which rebounds have been observed [78], [79]. In practice, whether IPT equates to chemoprophylaxis would depend on coverage, the timing of delivery and levels of drug resistance.

Limitations of the model components are discussed elsewhere [48]–[53], [80]–[84]. Some assumptions are especially relevant to this study. The predictions are likely to be sensitive to parameters relating to age-patterns and outcomes of severe disease in the first years of life. The predictions of indirect malaria mortality, and to a lesser extent, severe episodes rely on age-dependent co-morbidity functions. In a trial setting with access to good health care, the age-pattern of co-morbidity may be quite different to that assumed by our models, which were fitted to datasets from other settings [51]. This would affect age-dependent comparisons such as between IPTc and IPTi, and post-intervention effects. We acknowledge that a limitation of this study is the lack of a full sensitivity analysis. We are currently developing an interface which will facilitate extensive probability sensitivity analyses. Additionally, an ensemble of models with alternative assumptions where uncertainty exists would provide information on model sensitivity.

The model component for the action of antimalarial drugs was compatible with our within-host model. The drugs are assumed to act on the infection, either clearing or sparing it. This model would be unable to account for observed effects such as density-dependent cure rate or effects of acquired immunity. More sophisticated models for within-host parasite dynamics and drug concentrations are in preparation.

We used DALYs as an aggregate measure to minimise the number of predictions presented. They are an imperfect measure and depend on value judgements for the disability weighting, discounting and age-weighting and on the life table used [85], [86]. We followed standard practices, and calculated the DALYs with no age weighting recognizing that there is a lack of consensus on this issue [87], [88].

The simulations assume a low coverage of case-management and no other interventions. This is a common approach which measures the ‘full’ impact of an intervention and offers consistency when comparing ICERs. However, we recognise decision makers may already have a variety of interventions in operation and want to know the incremental benefit of changing their existing status quo. Model components for other interventions are under development and these predictions for IPT contribute to a growing database of the likely effectiveness of different malaria control strategies generated using a common simulation platform.

When considering IPTc or IPTi for a specific location, both the local characteristics and issues other than epidemiological impact and cost-effectiveness should be considered. This study does not address issues of affordability nor of safety, development of drug resistance, first line treatment drug choice, sustainability or malaria species other than *P. falciparum*.

In conclusion, modelling can extend the information available to policy-makers by providing predictions of the likely impact and cost-effectiveness for settings, for outcomes and for multiple strategies where, for practical reasons, trials cannot be carried out. Our predictions indicate that both IPTi and IPTc can be cost-effective interventions in a range of settings. This cost-effectiveness is driven by low delivery costs and the predicted impact on mortality. Both IPTi and IPTc could be rendered cost-ineffective by very low transmission, mis-timed seasonal delivery, ineffective drugs, very high treatment coverage or combinations of these factors. Seasonal delivery is more cost-effective in seasonal settings and year-round in constant transmission settings, the difference is more pronounced for IPTc than IPTi. Predictions suggested that the optimum target age group for IPT in settings with a very low transmission intensity would include children over five years.
